# Medical Miscellanies

**Published:** 1817-03

**Authors:** 


### PART IV.

### MEDICAL MISCELLANIES.

### THE PORTE FEUILLE, No. III.

OR,

DEPOT FOR DETACHED FACTS AND INTERESTING PHÆNOMENA, OCCURRING IN THE PRACTICE OF THE FRIENDS OF THE MEDICO-CHIRURGICAL JOURNAL.

"Ore trahit quodcunque potest atque addit acervo."

*Dissection of the Case of Extraordinary Disease of the Thigh, described in No. I.*

*External Appearances.* — The tumour of the thigh, instead of being elastic, tense, and circumscribed, as it had been during life, was now become flaccid, and more generally diffused over the limb. On the internal side, over the situation of the *triceps adductor femoris*, the cuticle was separated to a considerable extent, by fœtid and discoloured serum. A remarkable fullness of the integuments also shewed itself on the corresponding side of the body, from the *crista illii* nearly to the inferior angle of the scapula; and a similar separation of the cuticle had taken place there. The leg and foot were slightly œdematous. The right testicle felt greatly indurated and enlarged; and there was considerable effusion of gelatinous fluid in the reticular structure of the scrotum.

*Examination of the Thigh.* — An incision being made from the anterior superior spinous process of the ilium to the pelvic attachment of the *gracilis*, and another from that, straight down to the patella, in the course of the *rectus femoris*, the integuments were dissected back from about half the circumference of the limb, and the fascia exposed. Considerable quantities of straw-coloured serum oozed from the cellular membrane during this stage of the dissection. My attention was particularly arrested by the strong artery-like structure of the *vena saphena interna*, for the space of some inches before it dips beneath the fascia in its passage to the femoral vein. The vessel, on being divided, remained perfectly patent.* The whole tumour was now brought into view. Its immediate parietes were formed by the *glutæus maximus*, *tensor vaginæ femoris*, *Sartorius*, *rectus femoris*, *gracilis*, *triceps adductor*, and the other muscles not extensively attached to the bone. They were all pale, flabby, wasted, and unnaturally broad. The commonly narrow, and elegant *gracilis*, had acquired a width of nearly three inches. On its posterior border was seen the puncture made in the first and third operations. A quantity of serum issued from it on pressure. An incision was now directed into the tumour through its whole extent, on the anterior margin of the *gracilis*: and, on penetrating the fibres of the *triceps* in the same direction, an enormous cavity was exposed; from whence rushed forth a torrent of highly offensive fluid, in colour and consistence most accurately resembling *strawberries and cream.* A moderately sized chamber pot was filled with that part of the fluid which I could collect by means of a sponge. This, with the quantity spilt upon the floor, could scarcely have amounted to less than six pints. On examining the interior of this great cavity, I found the *vastus internus* and *externus*, the *cruræus*, and short head of the *biceps*, converted into a pale, soft, almost gelatinous mass, retaining little of their original bulk, and no appearance of muscular structure. My arm, passed under the thigh bone, reached with facility the superior boundary of the sac near the *crista ilii.* The bone itself, seemed perfectly sound except on its posterior surface just below the trochanter minor ; where for the space of about half an inch, it was destitute of periosteum, rough, and scabrous. Passing my hand along the shaft of the bone towards the knee, I laid hold of a somewhat firm and very irregular mass, apparently adhering to the internal aspect of the bone.† It was detached with difficulty ; and on removal, was found to consist of numerous pale, semi-transparent cysts or vesicles, clustered together like a bunch of

* This was probably attributable to long-continued compression. I remember, in another instance, to have seen the *vena saphena* in the same state, from having been long subjected to the pressure of a tight bandage employed with a view of curing a varicose state of the veins of the leg.

† Upon this point it will be recollected, the injury was first received; and here the pain and induration were constantly felt from the beginning. The impression on my mind, at the time, I confess, was that the mass consisted of a *cluster of hydatids*. Probably, I was mistaken. I regret that the opportunity of again examining the morbid structure was denied me. Summoned from the dissection to an accident, I laid the mass by, as I thought, in a place of security; but found, on my return, that with the fluid removed from the tumour, it had been consigned to a recess, from which all my ardour of research was insufficient to excite me to attempt its rescue.

grapes, but with margins much less clearly defined. The whole mass might, I conceive, have weighed from eight to ten ounces. I laid it aside for more minute and deliberate inspection, but my intention in this respect was unluckily frustrated.

All the *organs of the abdomen and thorax* were sound, except that the gall bladder contained one large, smooth, spherical concretion, weighing, when recently removed, 157 grains; and three or four very minute ones. The dissection of the limb detained us so long that the brain was not examined.

I know nothing that I can usefully add, by way of comment, on this extraordinary case. The dissection was witnessed by several professional gentlemen, to whose testimony I might, if it were requisite, appeal, in proof of the correctness of my description. An Army Surgeon, to whom I mentioned the case, assured me he had seen one of nearly a similar nature; but as I made no memorandum of our conversation at the time, I dare not trust to my recollection in detailing the particulars of it, which he then communicated. Should we meet again, I shall request him to give it me for your PORTE-FEUILLE, to which I purpose to become a frequent Contributor.

### *Abscess in the Lungs bursting outwards, and rendering carious the Ribs; with other remarkable Appearances.*

On the 26th of September, 1816, Dr. Johnson had an opportunity of opening a man who had died the preceding day of a lingering illness. No complete history of the case, however, could be procured. The first symptoms were those of enlarged liver, which viscus indeed could be plainly felt externally. Then followed cough, and pain in the right side of the thorax; but no expectoration. The left side of the chest now bulged out, so that the patient appeared crooked. A tumour formed over the sixth and seventh ribs of the right side; which bursting, continued to discharge ill-conditioned matter for five or six months; indeed till the day of his death. In the early period of this abscess, air was frequently expelled, when coughing, from the wounds; but, as will hereafter be seen, this air had probably entered by the same orifice. Finally, he emaciated and died.

*Sectio Cadaveris.* On opening the abdomen, an immense liver presented itself, very hard in its texture, especially at the edges, and encroaching on that side of the thorax as high as the sixth rib. It weighed eight pounds. Its structure appeared no otherwise altered than by induration. As it stretched up above the external opening of the abscess, we prejudged that the liver was the seat of the abscess; but this was not the case. The pancreas was scirrhous. The spleen was enlarged. Adherent to the latter viscus by some loose cellular substance were found two perfectly globular bodies, one the size of a small apple, the other of a small marble. They were, in substance, identically the same as the spleen itself. When the loose cellular membrane was stript off them, their surfaces were beautifully polished every where. They appeared, in fact, independent spleens.

In the thorax, the heart and pericardium were natural, except that they were pushed over entirely into the left side. The lung of that side was sound, and of great volume. The other lung was shrunk into a solid mass about the size of a small spleen, adhering firmly to the costal pleura. The liver, pushing up before it the diaphragm, usurped the space which the right lung had formerly occupied when sound. On opening the diseased lung we found it a mass of calcareous matter, cartilage, and a solid substance like indurated liver. The calcareous depositions appeared like broken pieces of arborescent coral, and lay partly loose, partly ramified through the other substances. No trace of air cells or bronchia could be seen; but the roots of the pulmonary artery and vein were partly ossified, partly cartilaginous. In the centre of this mass were the parietes of an abscess, the cavity of which still contained some fœtid matter. The ribs adjacent were more than half an inch in thickness. The communication of the external opening with the abscess was between the sixth and seventh rib.

Dr. Baillie has seen but one instance of partial ossification of the lungs, and there was a strong disposition to ossification in other parts of the system. " Earthy concretions," says he, " have occasionally been found in the lungs, although it is a rare appearance of disease." p. 76.

Bonetus quotes a case from *Miscell. curiosis anni* 1671, which bears some resemblance to the foregoing. " *Apertis pulmonibus eosdem hinc inde plurimis calculis millii magnitudinem æquantibus refertos invenimus, copiamque illorum ad duo cochlearia capacia colleginus.*" The patient never had pain or difficulty in breathing. Fabricius Hildanus (cent. ii. obs. 29.) found in a man who had died emaciated with cough, a hard stone in the substance of the lungs. " In pulmonis substantia lapidem prædurum inveni." " A man [*Sepulchret. Boneti de respiratione læsa obs. iii.*] without cough or expectoration laboured under great dyspnœa. " He was carried off by a fever. *Lapides varii, variæque figuræ, asperæ arteriæ incumbentes ita eam oppresserant ut tandem hominem præfocaverient.*"

The body of a young man was opened by Fallopius in the year 1561, who found four stones in the lungs, each as large a bean. " *In ejus pulmone quatuor lapides offendit, nullum faba majuscula minorem.*"

An Italian woman, 35 years of age, had long laboured under difficulty of breathing, with dry cough, loss of appetite, &c. of which she died. " Dissecto ejus cadavere ventriculi omnes fibræ, nigræ, duræque repertæ sunt: tres vero lobi pulmonum *adeo durati ut potius tophos lapideos quam carnem* omnibus videntibus ementirentur." *Obs. Petri Medici Florent. Sepulchret. Bonet. lib. 2. Sect. i.* Silvaticus also relates the case of a nobleman who died " ex asthmate gravissimo," whose lungs were so indurated —" *ut vitri cocti* instar frangerentur." *Sap. Boneti.*

Dr. W. B. S. a medical gentleman of some talents, had been for many years subject to dyspnœa and habitual cough, but not to regular paroxysms of asthma. In July 1815 ,he was afflicted with a severe anthrax on the back part of the neck, which required deep and extensive incisions. From that period his health was never re-established. Shortly after the carbuncle was healed, he noticed an unusual thirst, especially in the mornings. Towards the beginning of November, the thirst increased, and about this time his appetite became unusually keen. The urine was discharged more copiously and frequently than usual; but this was attributed entirely to the quantity of liquids taken in. Some slight degree of emaciation now took took place, but no alarm was excited till towards the beginning of March 1816, when a friend recommended him to examine his urine, and confine himself as much as possible to animal food. He had so little apprehension of diabetes, that he neglected to examine his water, and his complaint was considered a renal affection, and treated accordingly. About the end of March, the same medical friend called again, and found him making from fourteen to sixteen pints of straw-coloured urine. His tongue was white; appetite voracious; thirst insatiable; pulse 120; countenance shrunk. He had a dull pain in the region of the kidnies; and great irritability of temper. No doubt had existed in his friend's mind from the first, about the diabetic nature of the disease; but now, on tasting the urine, and finding it like honey, a communication was made to his friends; a consultation called, and the disease pronounced diabetes. Dr. Watt's plan was tried, principally from the patient's own choice. ℥xxv of blood were abstracted from the arm, the warm bath was employed, and some opening medicine exhibited. During the next twenty-four hours after the venæsection, the quantity of urine was increased, by two pints, and exceeded the whole of the ingesta. Such was the alarming debility that ensued, that but one more venæsection was tried;—he sunk rapidly, and expired on the 15th of April 1816.

This gentleman was about 48 years of age; yet the disease which carried him off, appeared a *break up* of the constitution, which no human power could check. The dyspnœa and pulmonary expectoration; the anthrax; and, lastly, the diabetes, were *means* by which Nature was mouldering away a fabric which she could no longer sustain.

*The Progress of the Vaccine Pustule arrested in consequence of Immersion in cold Water.*— Some years since, a healthy lad was inoculated with the fluid taken from a fine regularly formed cow-pox pustule. The usual signs of its having taken effect were visible on the third day. The disease went on favourably till the sixth. On that day, the lad was accidentally thrown from a horse into a pond of water; but sustained no injury, except a complete ducking, and the terror necessarily caused by such an occurrence. On the following day, the attendant surgeon examined the arm, and found, to his surprise, that *every trace of the incipient vaccine pustule had disappeared.* The lad was some time after again inoculated, and went regularly through the disease. Is the prompt disappearance of the cow-pox inflammation, in this case, attributable to the influence of sudden terror on the lad's mind, or to the physical operation of the cold immersion?

---

### MISCELLANEOUS.

Quicquid agunt Homines.

### *The OOPAS or POISON TREE of JAVA.*

( Communicated by B. W. Coley, Esq. of Cheltenham. )

" Soft zephyrs blow, eternal summers reign,
And show'rs prolific bless the soil—in vain!
No spicy nutmeg scents the vernal gales,
No tow'ring plantain shades the mid-day vales;
No *step retreating*, on the sand impress'd,
Invites the visit of a *second guest!*
Fierce in dread silence on the blasted heath
Fell Upas sits, the hydra tree of death!" *Darwin.*

The poetry of Darwin has, in some measure, consecrated the fiction of Foersch, whose daring imposition on public credulity has too long remained without refutation. But although the situation of the poison tree; the application of the Oopas; the mode of collecting it, &c. in the Dutch writer's account, be fabulous; yet the existence of a tree on Java, from whose sap a poison is prepared, equal in fatality, when thrown into the circulation, to the strongest animal poisons hitherto known, is a fact, which is fully established by Dr. Horsefield in his Report to Lieutenant Governor Raffles, of Batavia; published in the 7th Volume of the Transactions of the Batavian Society.

The tree which produces this poison is called Antshar, and grows on the eastern extremity of the island. The stem is cylindrical, perpendicular, and rises to the height of sixty or eighty feet, completely naked. It is covered with a whitish bark, slightly bursting in longitudinal furrows, which being wounded near the ground, yields plentifully the milky juice from which the oopas poison is prepared. The consistence is nearly that of milk, only somewhat thicker and more viscid. The juice is contained in the cortex, from a single puncture in which, a cupful may be collected if the tree be large. The tree delights in a fertile, and not very elevated soil, and is only found in the largest forests. It is with difficulty the inhabitants can be made to approach the Antshar, from a dread of the cutaneous eruptions which it produces when newly cut down. But excepting when the tree is largely wounded, and the atmosphere impregnated with the effluvia of the juice disengaged, it may be approached and ascended like the other common trees in the forest. "In no instance," says Dr. H. "have I observed the ground naked or barren in its immediate circumference. The largest tree I met with in Blambangan was so closely environed by the common trees and shrubs of the forest, that it was with difficulty I could approach it. At the time I visited the tree and collected the juice, I was forcibly struck with the egregious misrepresentation of Foersch. Several young trees sprung from seeds that had fallen from the parent, reminding me of a line in Darwin.

"Chained at its root two scyon demons dwell."

While in recalling his beautiful description of the oopas, my vicinity to the tree gave me reason to rejoice that it is founded on fiction."

Besides the antshar, Java produces a shrub called the TSHETTIK, furnishing a poison still more violent and fatal than that of the former. The larger tshettiks have a diameter of two or three inches, covered with a reddish brown bark, containing a juice of the same colour, of a peculiar pungent, somewhat nauseous odour. It is from the bark that the poison is prepared; a decoction of which is thickened by evaporation to the consistence of syrup, after which the preparation is the same as of the antshar now to be described.

"The process of preparing the antshar was performed for me by an old Javanese, who was celebrated for his superior skill in preparing the poison. About eight ounces of the juice of the antshar, which had been collected the preceding evening, in the usual manner, and preserved in the joint of a bamboo, was carefully strained into a bowl. The sap of the following substances which had been finely grated and bruised, was carefully expressed and poured into it, viz: arum, nampoo, (Javanese) kæmferia, galanga, kousthur, amomum, bengley, (a variety of zerumbed) common onion and garlic, of each about half a drachm; the same quantity of finely powdered black pepper was then added, and the mixture stirred. The preparer now took an entire fruit of the capsicum fructicosum or Guinea pepper, and having opened it, he carefully separated a single seed, and placed it on the fluid in the middle of the bowl. The seed immediately began to reel round rapidly, now forming a regular circle, then darting towards the margin of the cup, with a perceptible commotion on the surface of the liquor, which continued about one minute. Being completely at rest, the same quantity of pepper was again added, and another seed of the capsicum laid on as before; a similar commotion took place in the fluid, but in less degree, and the seed was carried round with diminished rapidity. The addition of the same quantity of pepper was repeated a third time, when a seed of the capsicum being carefully placed in the centre of the fluid, remained quiet, forming a regular circle about itself; in the fluid resembling the halo of the moon: this is considered as a sign that the preparation of the poison is complete."

Of twenty-six experiments detailed by Dr. Horsefield, we shall only extract four, as illustrative of the effects of these remarkable poisons; the operations of which, on the animal system, are essentially different.

The common train of symptoms is a trembling and shivering of the lower extremities, restlessness, discharges from the bowels, drooping and faintness, slight spasms and convulsions, hasty breathing, an increased flow of saliva, spasmodic contractions of the pectoral and abdominal muscles, retching, vomiting, great agony, laborious breathing, violent and repeated convulsions, death. The effects on quadrupeds are nearly the same, whatever be the site of the wound. To dogs, the oopas generally proves mortal within the hour. A mouse died in fifteen minutes; a monkey in seven minutes; a cat in fifteen minutes. A buffalo in two hours ten minutes. Birds are very differently affected by this poison. Fowls resist its effects strongly; some even recovered from the effects. The operation of the TSHETTIK is far more violent and rapid than that of the ANTSHAR. The *latter* exerts its influence chiefly on the stomach, alimentary canal, the respiration and circulation:—The *former* is determined to the brain and nervous system. After some symptoms of faintness, drowsiness, and convulsions, the TSHETTIK acts by a sudden impulse, which, like a violent apoplexy, prostrates at once the whole nervous system.

In animals killed by the ANTSHAR, the large vessels in the thorax, the aorta and venæ cavæ, were, in every instance, found excessively distended. The surrounding viscera, especially the lungs, were gorged with blood of a florid colour, and completely oxygenated. When the vessels were punctured, the blood sprang out as if from living vessels. The stomach was usually distended with air; but the brain was less indicative of morbid effects than the viscera of the thorax and abdomen. In some instances it was perfectly natural—in others, slightly inflamed.

The post mortem appearances in animals destroyed by TSHETTIK were very different from the above. The viscera of the abdomen and thorax were found nearly in a natural state, as were the great vessels leading to and from them. The brain and dura mater, on the contrary, exhibited marks of violent action. In some instances, the inflammation and redness of the dura mater were so strong, that on first inspection Dr. H. supposed them to be the consequence of a blow previously received, until he found, by repeated examinations, that these were universal appearances after death by *tshettik.*

Rumphius, who has published on this poison, and who had an opportunity of personally observing the effects of the poisoned darts or arrows on the human system, as they were used by the natives of Macassar, in their attack on Amboyna, about the year 1650, says, that the poison touching the warm blood, is instantly carried through the whole body, so that it may be felt in all the veins, and causes an excessive burning, and violent turning in the head, which is followed by fainting and death. After having proved mortal to many of the Dutch soldiers in Amboyna and Macassar, they are said to have finally discovered an almost infallible remedy in the root of the crinum asiaticum (radix toxicaria) which, if timely applied, counteracted by its violent emetic effect, the force of the oopas.

An intelligent Javanese informed Dr. Horsefield, that an inhabitant was wounded in a clandestine manner by a poisoned arrow in the forearm, near the articulation of the elbow. In about fifteen minutes he became drowsy, after which he was seized with vomiting, became delirious, and in less than half an hour died.

*Experiments by Dr. Horsefield.*

1. A dog of middling size, was wounded in the muscles of the thigh by an arrow that had been immersed into the newly prepared oopas, and which had been exposed to the air one night. In three minutes he seemed uneasy, and in twenty-five minutes he fell down suddenly, screamed, extended his extremities convulsed, discharged his excrements; the froth falling from his mouth. On the twenty-sixth minute he died.

*Dissection.* The abdomen being opened, about five minutes after death, a small quantity of serous fluid was found in the cavity; the liver, intestines, and other viscera were natural. In the stomach a yellowish frothy mucilage was found adhering to the internal coat, which was contracted into wrinkles. In the thorax the lungs were of an elegant florid colour, and gorged with blood; the pulmonary vessels exhibiting through their coats a florid sanguinary fluid; on puncturing the ascending aorta the blood gushed out of a florid colour. In the venæ cavæ the blood was of the usual dark hue, and on a puncture flowed out forcibly; the muscles of the extremities were remarkably pale; on tracing the wound, it was found inflamed, and in two places along its course, a small quantity of blood was found effused between the muscle and tendon.

2. A small dog was wounded in the muscles of the thigh with the simple unprepared milk of the antshar. From the moment of the puncture he continued barking and screaming incessantly eight minutes; he now extended his tongue, locked his jaws, was seized with twitchings of the extremities and with convulsions of the abdominal muscles, and discharged the contents of the bowels. On the tenth minute he sprung suddenly and barked violently, but soon became exhausted, and laid down quietly on the ground. On the twelfth minute he fell prostrate, was convulsed; after which having lain apparently motionless one minute, the convulsions recurred with greater force; on the fourteenth minute he died. On dissection all the vessels in the thorax were found excessively distended with blood. In the abdomen the stomach was almost empty, but distended with air, and its internal coat covered with froth. The vessels of the liver were gorged with blood.

3. The following experiment was made on an animal of the ox tribe in common domestic use in Java, called korbow by the Javanese, and *buffalo* by the Europeans: the subject was full grown and in perfect health. Having being well secured, he was wounded by a dart, somewhat larger than those used in the other experiments, covered with the oopas from Blambangan (applied about twenty-six hours before) into the internal muscles of the thigh, in an oblique direction, the skin having been previously divided to admit the weapon freely. The animal being in some degree loosened, about one minute after the puncture the dart was extricated. I suppose that about six grains adhered to the wound; on the tenth minute, the respiration was somewhat increased and heavy. In twenty minutes, he had a copious discharge from the intestines, a watery fluid flowed from his nostrils, and he showed some symptoms of drowsiness. In thirty minutes he had an increased flow of saliva, which dropped from his mouth; he extended his tongue, and licked his jaws; his respiration became more laborious; his pectoral muscles acted with violence, and the abdominal muscles were strongly contracted above the pelvis. His motions were slow and difficult, his muscular exertions were much diminished, and he exhibited great fatigue, accompanied by restlessness: all these symptoms gradually increased. The sixtieth minute, his hair now stood erect; unable to support himself, he lay down; he had spasms and contractions of the extremities; the abdominal muscles were more violently convulsed, and the respiration more laborious. The restlessness rapidly increased; having risen with difficulty, he quickly lay down again, exhausted and panting; the flow of saliva from his mouth continuing. In seventy-five minutes he extended his tongue and made an attempt to heave, his extremities trembled; he rose and threw himself down again suddenly, extending his head. On the eightieth minute, the saliva flowed in streams from his mouth, mixed with froth; he heaved violently with excessive convulsive actions of the pectoral muscles; but unable to vomit, he appeared in great agony. In ninety minutes he extended his head with strong convulsions, trembled; the hair stood erect, he discharged the contents of his bowels, his breathing became more laborious, and the muscles of the abdomen and breast acted with excessive violence. The agony increasing, he rose for a few seconds; but unable to support himself, fell down again. The hundred and tenth minute, having made an attempt to rise, he fell down head foremost with convulsions of the extremities and head; he groaned violently; the respiration was much impeded, and recurred at intervals of fifteen seconds. On the hundred and twentieth minute, he lay in great agony, groaned, bellowed, and extended his tongue and extremities, violently convulsed. In a hundred and twenty-five minutes he was entirely exhausted; the breathing returned after long intervals. On the hundred and thirtieth minute, he died convulsed. Fifteen minutes after the motions of life had ceased, I opened the cavity of the abdomen, and exposed to view the contents of the breast. The stomach was immensely distended with air, the vessels of all the viscera of the abdomen were as if injected and distended with blood. In the thorax the lungs were of a vivid, florid, crimson colour, and the great vessels, both the aorta, and venæ cavæ, and the arteries and veins of the lungs were gorged with blood. A small puncture being made into the aorta, the blood bounded out in a stream, of a beautiful crimson colour; from the venæ cavæ it flowed of a dark livid colour. In the large muscles of the pectus, which had been divided in the dissection, a trembling vibratory motion was perceived full twenty minutes after the motions of life had ceased.

*Experiment with Tshettik.*

4. A dog, of the middling size, was wounded in the muscles of the thigh with a dart covered with the fresh prepared poison of tshettik. In two minutes he shewed symptoms of uneasiness; he appeared faint and lay down. On the third minute, he was seized with convulsive twitches of the extremities, was very restless, and his breathing became quick; these symptoms gradually increasing to the sixth minute, while he continued as exhausted in a lying posture. He now raised himself, extended his head as if attempting to leap, but fell down, was seized with violent convulsions, attended by quick and interrupted breathing to the ninth minute, when he died.

The appearances on dissection, corresponded with what has been stated in the beginning of this paper.

---

*On Cerebral Inflammations as determined by Affections of the Digestive Organs; by M. Lespagnol.* — Our brethren in France are beginning to see the strong connection between the head a d epigastric viscera, and their reciprocal influence exerted on each other, both in functional and structural diseases. The valuable writings of Cheyne, Abernethy, Curry, Yeates, and some other authors, however, seem to be little read or understood in France. The observations of M. Lespagnol are drawn principally from among children, where the gastro-cerebral sympathy is more evident than in those of a more adult age.

Case 1. A lad, 12 years of age, and slender, was seized on the 18th of May, with fever, accompanied by headache, nausea, and vomitings. 19th. A vomit was exhibited, after which, the headache was intense. He had now epigastralgia; dry cough; tongue red; skin hot; pulse quick and strong; countenance depressed; voice sharp. On the night of the 21st, he was harassed with vomitings. 22d. More tranquil; at night much distressed, but the vomitings not so frequent. 23d. Features contracted; lips red, and chapped; tongue rough in the middle; mouth half open; thirst insatiable; violent epigastralgia; sense of burnning heat in the abdomen; mucous vomitings, every time he drinks; anxiety; constant jactitation, and attempts to throw the clothes off the bed; obstinate constipation since the day after the emetic was taken; the pulse is frequent but weak; respiration quick; intellectual faculties clear. In two hours, the symptoms assumed a tone of nervous debility. The pain and sensibility about the stomach disappeared, and symptoms of phrenitis only were observable. Sinapisms to the feet had no effect. Four leeches to the neck procured but a momentary relief. The application of ice to the head brought on convulsions. A purgative enema brought away copious stools, but without alleviation of the complaint. In the course of the day there appeared a kind of alteration of cerebral and abdominal inflammation; but the former prevailed, and death closed the scene about midnight.

*Sectio Cadaveris.* A puriform infiltration in the cellular tissue of the pia mater, throughout its whole extent. Traces of inflammation in the tunica arachnoides. A flattening of the circumvolutions; the substance underneath soft, and injected. A small quantity of water in the ventricles of the brain. The larynx, pharynx, and thoracic viscera sound. The borders of the cardiac orifice of the stomach very much inflamed. The pyloric orifice slightly reddened. The whole mucous membrane of the stomach dotted with red points. The small intestines of a violet colour externally, and their vessels very much injected. Red streaks here and there on the great intestines. The epiploon inflamed. The liver very large, and gorged with blood. The urinary organs slightly inflamed.

M. Lespagnol thinks, with probability, that the visceral inflammation of the abdomen must have commenced anterior to that of the brain. A state of cerebral inflammation, capable of inducing such extensive disease in the abdomen, would have assumed a graver aspect in the beginning, and would have destroyed life in a shorter time. Our author justly observes, that the emetic exasperated the visceral inflammation, and that a propagation of the affection to the head was the result. On the *methodus medendi* we need not remark further than that the "*medicine expectante*" is a dangerous remedy in acute diseases of any kind, but in gastro-cerebral affections in particular.

M. Lespagnol next applies the gastro-cerebral sympathy to the formation of hydrocephalus, which he believes to be frequently produced by chronic inflammation in the abdomen, and the intestinal irritation of worms.

*Morbid Anatomy.*—In a cyst removed from the anterior margin of the liver of a woman, aged 70, Mr. Laugier has recently found the contents consisting at most wholly of *phosphate of lime.* The envelope of the cyst was thick and fawn coloured. Through the inclosed pulpy substance were interspersed many solid, hard concrete lumps. Portions of the cyst, digested in warm alcohol, yielded a substance resembling the adipocire of biliary calculi; which was deposited, on the cooling of the fluid, in the form of lamellar crystals; but in such a minute quantity that its precise characters could not be ascertained. *Annales de Chimie et de Physique, Juin,* 1816.

*Preservation of Sulphuric Ether.*—The purest sulphuric ether becomes acid after a certain time, when the vessel, containing it, is not completely full, and when it is frequently exposed to the influence of air and light in a somewhat elevated temperature. Hence, the apothecary should keep his stock of ether in the cellar, and as much as possible, in full vessels; having only a small quantity in the shop for daily use. *Annales de Chimie et de Physique. Juin*, 1816.

*Poisoning by Mushrooms.*—A French writer asserts that sulphuric ether, administered in large doses, either alone or in combination with syrup or honey-water, will remove the symptoms consequent on poisoning by mushrooms, even in their most violent form. *Gazette de Santé. Juillet*, 1816.

*Obscure Lesions of the Stomach.*—M. Pinel has observed in many persons who have died from diseases not implicating the stomach, red spots on the mucous membrane of the organ, varying in size, and sometimes of so deep a colour, as to excite suspicion of inflammation, which, however, had not existed during life. In females addicted to spirituous potation, he has frequently discovered numerous small ulcerations of the stomach and intestines, without decided inflammation. Professor Chaussier has, many times, seen in women recently delivered, erosions or perforations of the stomach; the existence of which could not have been previously suspected. This remarkable fact should constantly be borne in remembrance by the juridical physician and surgeon. *Vanderschilt. Journal de Médecine. Par Leroux. Juillet*, 1816.

*Preservation of Milk.*—Kirchoff, the chemist, to whom we owe the discovery of forming sugar from starch, has employed a process, more than a century since described in the *Memoirs of the Leopoldine Academy of Vienna.* Fresh milk is slowly evaporated by a very gentle heat, till it is reduced to a dry powder. This substance should be kept in a dry and well stopped bottle. Diluted with a sufficient quantity of water, it possesses considerable analogy, both in its taste and other properties, with new milk. Eggs may be preserved in a similar manner. *Gazette de Santé. Aout*, 1816.

*Amputation successful in the Eighth Month of Pregnancy.*—M.B. ætat. 24, was severely wounded in the lower extremities by the bursting of a shell, on the 23d July, 1815. There was an extensive and deep contused wound on the inside of the left leg; the extremity of the tibia fractured; the ankle joint exposed; and the astragalus shivered. On the right leg was a severe wound of the soleus muscle; rupture of the tendo Achillis, and division of the posterior tibial artery. The latter vessel was immediately secured, and the wound dressed with proper attention to the divided tendon. In respect to the left leg, it was evident that nothing could save it. The woman declared herself in the eighth month of pregnancy, but amputation was determined on, and immediately performed by

M. Nicod, in the hospital of Beaujon. The fever was very great on the evening of the first day, but gradually subsided, and totally disappeared on the fourth. On the sixth and seventh days, some suppuration appeared; and on the evening of the latter, a very intense fever, preceded by shiverings and pains in the abdomen. On the eighth day, the fever became remittent, and preserved this type till the fourteenth day. On the thirteenth day, the amputation ligatures came away; and the cinchona bark was exhibited. By the twenty-second day, the fever was stopped. The wounds of the left leg became very troublesome with extensive inflammation and abscesses. On the thirty-fourth day, she was partureal, and next day was delivered of a fine living child. At this period the left leg was in a very wretched state; nevertheless, she gradually recovered, and was able to go on a wooden leg in three months from the amputation. *Bulletin de la Faculté, &c. No.* 8, *Septembre,* 1816.

*Extract of a Letter from Mr.* H. W. Bailey, *dated Thetford, Feb.* 13, 1817.

"From the excessive wetness of last, and beginning of this month, together with the inferior quality of the bread, fevers of a low kind have been very prevalent among the poor. Whole families have been attacked with synochus, which have rapidly run into the typhoid type, and resisted every effort of the medical practitioner. Several have terminated fatally. A case of ascites occurred in an old man, who had been subject to diabetes several years, and which was completely suspended during the dropsical disease: it terminated fatally. In one patient, chorea sancti viti succeeded acute rheumatism. A young gentleman, of delicate habit, became affected with acute rheumatism, which in the first instance was treated on the antiphlogistic plan, v. s. aperients and saline medicines. The swellings of the joints and pain were extremely violent. In the course of ten days, the swellings subsided, and pain had left the joints; the tongue became clean, pulse soft and equal, bowels regular, and appetite returning. Bark and generous diet were enjoined, and a rapid recovery was apparently taking place. This was succeeded by an involuntary action of the limbs and muscles of the face; the pulse extremely rapid, 160 in a minute; debility excessive; no pain; tongue white. During this distressing state, an able physician was consulted, who prescribed tonics, nutritious diet, &c. which in three weeks completely restored him to perfect health."

A very interesting Work on Military Surgery, illustrated by numerous cases, is in an advanced state of preparation for the press, by Deputy Inspector of Hospitals Hennen. The Editors have reason to believe that the above work will prove as important an addition to Medical and Operative Surgery, as has appeared in modern times.

A Translation of Professor Weidman's Work on Necrosis is preparing for the press, by Robert Knox, M. D.

Dr. Burrows intends publishing, in a few days, "Cursory Remarks on a Bill, now pending in Parliament, for Regulating Mad Houses throughout Great Britain; and upon its probable influence on the Physical and Moral Condition of the Insane, and upon the interests of those concerned in their care and management."

A Medical Student at Paris is now undergoing prosecution before the Criminal Tribunal, for having procured a Fellow Student to write his Thesis for him.—*Literary Gazette.*

The death of Mr. Royston, who had been nominated to deliver the next Anniversary Oration, on the 8th of March next, before the Medical Society of London, has occasioned some difficulty to the Council, to obtain, at so short a notice, a substitute. After much pressing entreaty, Mr. Stevenson, of Great Russell Street, we are informed, has at length consented to undertake the arduous task.

Deaths.—At Maidstone, aged 83, Sir William Bishop, M. D. At Calcutta, Charles Besborough, Esq. of the Honourable East India Company's Medical Establishment. At Leeds, Joshua Walker, M. D. Mr. John William Korb, late Surgeon of the 49th Regiment.

